# Association between ADHD symptoms and device‐measured physical activity and sedentary behavior in childhood: A population‐based twin study

**DOI:** 10.1002/jcv2.70141

**Published:** 2026-06-09

**Authors:** Narda Ontiveros, Björg Helgadottir, Örjan Ekblom, Anna Ohlis, Aylin Mehren, Camilla A. Wiklund

**Affiliations:** ^1^ Department of Health Sciences The Swedish School of Sports and Health Sciences (GIH) Stockholm Sweden; ^2^ Department of Neurobiology, Care Sciences and Society Division of Nursing Health Promotion Among Children and Youth Karolinska Institutet Huddinge Sweden; ^3^ Department of Global Public Health Karolinska Institutet Huddinge Sweden; ^4^ Department of Psychiatry and Psychotherapy University Hospital Bonn Bonn Germany; ^5^ Department of Medical Neuroscience Donders Institute for Brain Cognition and Behaviour Radboud University Medical Centre Nijmegen the Netherlands

**Keywords:** accelerometry, ADHD, physical activity, twins

## Abstract

**Background:**

Attention‐deficit/hyperactivity disorder (ADHD) symptoms are associated with adverse health outcomes; this may be partially due to unhealthy lifestyle factors such as low physical activity (PA) and high sedentary behavior (SED). However, this association remains unclear. ADHD and PA have a medium‐to‐high heritability; therefore, genetically informed studies are warranted. This study aimed to increase the understanding of the association between ADHD symptoms and time spent in PA and SED in children. Additionally, we aimed to determine whether sex, symptom type, and genetics influence this association. This could guide the design of health promotion and preventive interventions.

**Methods:**

This cross‐sectional twin study included 9‐year‐old children from the Swedish Twin Registry born from 2011 to 2015 who had complete data on ADHD symptoms and accelerometry. ADHD symptoms were assessed through a parental‐report questionnaire. Inattention and hyperactivity/impulsivity symptoms were analyzed together and separately. Triaxial accelerometry measured the average time (minutes) spent in SED, light physical activity (LPA), and moderate‐to‐vigorous physical activity (MVPA). Linear generalized estimating equations regression models were used to assess the association in the full cohort and stratified by sex. Within‐twin pair analyses were performed separately for dizygotic and monozygotic twins. Regression coefficients and 95% confidence intervals (CI) were calculated.

**Results:**

1966 children (52.8% girls) were included. In the full cohort, ADHD symptoms were significantly associated with less time in SED (*β* = −0.32; 95% CI ‐0.59, −0.06) and more time in MVPA (*β* = 0.30; 95% CI 0.12, 0.47). These associations were primarily driven by hyperactivity/impulsivity symptoms and were significant only in girls. The associations remained in dizygotic twins but became non‐significant among monozygotic twins.

**Conclusion:**

Our findings suggest that in girls, higher hyperactivity/impulsivity symptoms are associated with higher PA levels. Associations were largely explained by shared familial factors, including genetics. Longitudinal studies are needed to corroborate these results.

## Key Points

**What's known?**

- ADHD symptoms are associated with adverse health outcomes which may be partially due to low physical activity (PA). However, the association between ADHD symptoms and time spent in PA and SED remains unclear.

**What's new?**

- ADHD symptoms were positively associated with less time in SED and more time in MVPA. These associations were mainly driven by hyperactivity/impulsivity symptoms and only significant in girls.
- The associations were largely explained by shared familial factors, including genetics.

**What's relevant?**

- This provides new insights into the association, highlighting the importance of having health‐promoting and preventive interventions early in life that consider factors like sex and type of ADHD symptoms.
- Further research is needed to explore a potential genetic overlap between ADHD symptoms and PA.

## INTRODUCTION

Attention‐Deficit/Hyperactivity Disorder (ADHD) is a prevalent neurodevelopmental condition characterized by persistent symptoms of inattention, hyperactivity, and impulsivity that affect functionality in several life domains (Posner et al., 2020). ADHD diagnosis is associated with adverse health outcomes during adulthood, including a higher risk of obesity, mental comorbidities, and cardiovascular diseases (Chaulagain et al., 2023; Li et al., 2022; Lorenzo et al., 2021). Having ADHD symptoms in childhood has been associated with poorer physical health outcomes during midlife (Stott et al., 2026). Furthermore, genetic studies indicate that subclinical presentations of ADHD are also associated with a higher risk of cardiometabolic diseases (Du Rietz et al., 2024). It has been suggested that children with ADHD engage more in poor lifestyle factors (Holton & Nigg, 2020), thus, studying modifiable lifestyle factors that could mediate these negative health consequences is warranted.

Physical activity (PA) and sedentary behavior (SED) are lifestyle factors associated with many health outcomes. Studies based on parental and self‐reported PA suggest that children with an ADHD diagnosis spend less time in PA, more time in SED, participate less in organized sports, and have lower PA in adolescence compared to those without ADHD (Holton & Nigg, 2020; Khalife et al., 2014; Kim et al., 2011). In contrast, studies using device‐measured PA found that children with an ADHD diagnosis spend more time in PA than those without ADHD (Lin et al., 2013; Sempere‐Tortosa et al., 2021; Villalba‐Heredia et al., 2022). These inconsistent findings may indicate either that the associations are sensitive to methodology, population‐specific, or relatively weak, and therefore require further investigation. Methodological differences may be an important contributing factor, as prior studies have reported low agreement between self‐reported and device‐measured PA (Dyrstad et al., 2014; Skender et al., 2016). Despite growing interest in device‐measured PA, large‐scale studies using devices (e.g., accelerometry) to explore the association between ADHD symptoms and time spent in PA and SED in children remain scarce.

Most of these studies, examining the relationship between ADHD, PA, and SED, have primarily focused on clinically diagnosed children, conceptualizing ADHD as a binary phenotype. However, growing evidence indicates that psychiatric symptoms are continuously distributed in the population, with diagnosed cases representing the extreme end of a broader spectrum (Lahey et al., 2022; Taylor et al., 2018). Consistent with this perspective, a genetic link has been found between the extreme and subthreshold presentations of ADHD symptoms (Larsson et al., 2012; Stergiakouli et al., 2017). Therefore, restricting analyses to children with an ADHD diagnosis may overlook meaningful variability within subclinical presentations. Moreover, this could introduce selection bias, as receiving a diagnosis is influenced by factors like access to healthcare and socioeconomic status. Consequently, a dimensional approach may provide a more comprehensive understanding of the associations. Yet, dimensional evidence using general population samples remains limited, with one study reporting that higher hyperactivity symptoms in childhood predict more PA during adolescence (Selinus et al., 2021).

This study aimed to increase the understanding of the association between ADHD symptoms and accelerometry measurements of PA and SED in children. Furthermore, boys and girls can have different manifestations of ADHD symptoms and PA (Babinski, 2024; Brazo‐Sayavera et al., 2021; Mowlem et al., 2019), but there is limited knowledge regarding the role of sex and type of symptoms in this association. Thus, we additionally aimed to elucidate whether these associations differ by symptom type and sex. Given the medium‐to‐high heritability of ADHD, PA, and SED (Carlsson et al., 2006; den Hoed et al., 2013; Faraone & Larsson, 2019; Gielen et al., 2014), we aimed to explore whether shared genetic factors influence these associations using within‐twin‐pair analyses. A better understanding of these associations can help identify subgroups that might benefit more from health‐promoting and preventive interventions.

## MATERIALS AND METHODS

### Data sources

**FIGURE 1:**
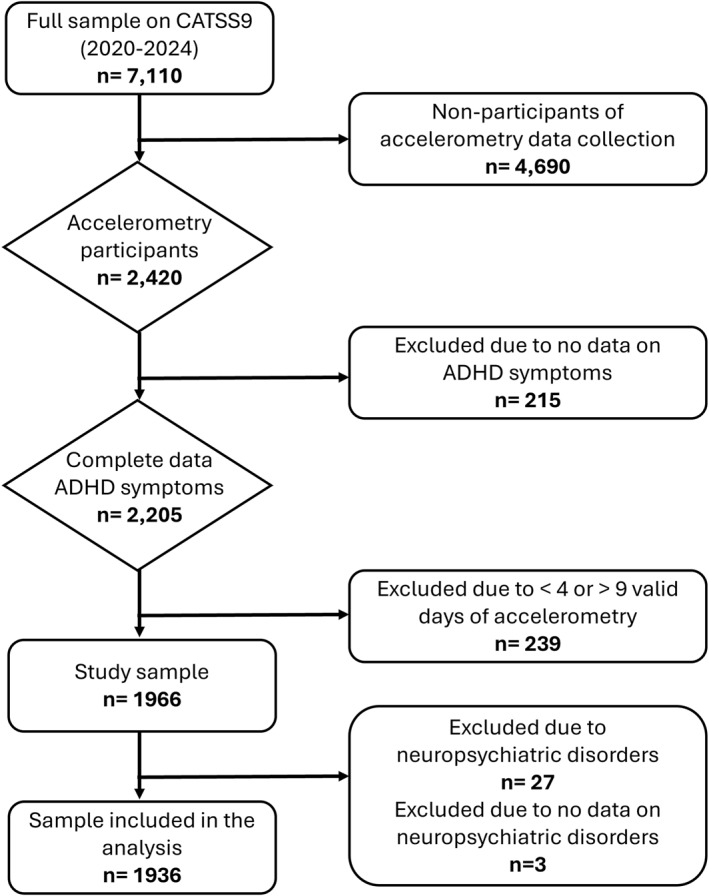
Flow diagram illustrating the study sample's inclusion and exclusion criteria.

The study population of this cross‐sectional twin study is drawn from the Child and Adolescent Twin Study in Sweden (CATSS) cohort. CATSS is an ongoing nationwide study that recruits twins born in Sweden from 1992 onward. The data collection at age nine (CATSS9) includes questionnaires regarding the twins' mental and physical health (Anckarsäter et al., 2011; Zagai et al., 2019). Since 2020, children participating in CATSS9 have also been invited to undergo additional PA measurements using accelerometry. Between 2020 and 2024, 7110 children participated in CATSS9, of whom 2420 also participated in the accelerometry data collection. Inclusion and exclusion criteria for the study sample are detailed in Figure 1. This study follows the Strengthening the Reporting of Observational Studies in Epidemiology (STROBE) reporting guidelines (von Elm et al., 2008).

### ADHD symptoms

The level of ADHD symptoms was parent‐reported using the Swedish translation of the DSM‐5 ADHD Symptom Checklist. This 18‐item questionnaire has not been formally validated, but it was designed to align closely with the DSM‐5 criteria. The items are divided into inattention and hyperactivity/impulsivity symptoms and are rated on a four‐point Likert scale (0 = Not at all, 1 = Just a little, 2 = Often, and 3 = Very often). The total ADHD symptoms score ranges from 0 to 54, and the subscale scores (inattention and hyperactivity/impulsivity) range from 0 to 27. These were analyzed as continuous variables. To meet the DSM‐5 criteria, a child must have at least 6 symptoms scored as “often” or “very often” (scored 2 or 3) on the inattention or hyperactivity/impulsivity items, or both. For sensitivity analyses, we used this criterion to classify children as having ADHD symptoms below/above the clinical threshold.

### Physical activity and sedentary behavior

Triaxial accelerometry (Actigraph GT3X) was used to measure SED and PA‐including light physical activity (LPA), and moderate‐to‐vigorous physical activity (MVPA). Children were asked to wear the accelerometer on the right hip for 7 days during their awake time, except during water‐based activities. Data were sampled at 30 Hz, using 3‐s epochs, and processed using Actilife (V.6.13.3). Non‐wear time was defined as 30 min of consecutive zeros with no allowance for interruptions (Vanhelst et al., 2019). A valid day was defined as having ≥600 min of wear time (Rich et al., 2013; Sievänen & Kujala, 2017); children with at least four and up to nine valid days were included in the current analyses (Migueles et al., 2017). The Romanzini cut‐off points were used to classify SED, LPA, and MVPA, which were developed against indirect calorimetry, giving a 0.93–0.99 ROC‐AUC (Romanzini et al., 2014). Average daily minutes spent in SED, LPA, and MVPA were analyzed as continuous variables.

### Other covariates

Sex and neuropsychiatric disorders were included as covariates. Sex was treated as a binary variable (boys/girls) and was defined as “sex assigned at birth”. Neuropsychiatric disorders were treated as a binary variable (yes/no), and it was defined as having a psychiatric or neurological diagnosis other than ADHD. The diagnoses included were autism, anxiety, cerebral palsy, chronic motor/vocal tics, depression, developmental coordination disorder, dyslexia, eating disorders, and obsessive‐compulsive disorder. The twins' zygosity was determined using a panel of 48 single‐nucleotide polymorphisms. If DNA samples were unavailable, a five‐question algorithm on twin similarity was used (Magnusson et al., 2013).

Maternal education level and parental‐reported PA were variables used in the sensitivity analysis. Maternal education level was categorized into elementary school (<10 years), upper secondary school (10–12 years), and university (>12 years). Parent‐reported PA included two questions: (1) *How much does your child move?* and (2) *Does your child exercise or play sports in his/her free time?*

### Data analysis

The descriptive characteristics of the study population were summarized by sex. Categorical variables were summarized as frequencies and percentages, and continuous variables were summarized as means and standard deviations. Sex differences were examined using Chi‐square and Wilcoxon signed‐rank tests, and regression models were used to evaluate the association between covariates and time spent in PA and SED.

To assess the association between ADHD symptoms and time spent in PA and SED, we performed linear regression models in the generalized estimating equations (GEE) framework with a robust sandwich estimator. This method does not make assumptions about data distribution and can account for the clustered nature of twin data. Analyses were performed on the full cohort, regardless of zygosity or sex. Three GEE regression models were estimated for each SED, LPA, and MVPA. Model 1 adjusted for accelerometry wear‐time, and Model 2 was further adjusted for sex. Model 2 was initially planned to be also adjusted for neuropsychiatric disorders, but due to their low prevalence (*n* = 27), they were excluded from all analyses. Model 3 additionally included an interaction term between sex and ADHD symptoms. Analyses were initially conducted using total ADHD symptom scores and subsequently repeated for inattention and hyperactivity/impulsivity symptoms separately. When a statistically significant interaction was observed, sex‐stratified analyses were conducted using Model 1. Regression coefficients, 95% confidence intervals (CI), and *p*‐values were calculated. Because SED, LPA, and MVPA are interrelated, we applied a Bonferroni correction to account for multiple comparisons. The adjusted significance threshold was set at *α* = 0.05/3 = 0.016. This correction was applied to the primary analyses. Interaction tests and stratified analyses were considered secondary and reported using unadjusted *p*‐values (*p* < 0.05).

To investigate genetic influences, we performed within‐twin pair analyses. Conditional GEE regression models were fitted separately for dizygotic (DZ) and monozygotic (MZ) twins. For this analysis, only MZ and same‐sex DZ twins were included. This analysis correlates the difference in ADHD symptoms within the pair with the difference in time spent in PA and SED, which implicitly controls for shared environmental and genetic factors. Because DZ twins share 50% of their DNA and MZ twins 100%, if an association observed in the full cohort persists in DZ but not MZ pairs, we can assume a genetic influence (Gonggrijp et al., 2023). Simultaneously, if the association remains significant among MZ twins, we can assume a true, possibly causal relation.

To assess non‐response bias, we conducted a sensitivity analysis, including children with data on ADHD symptoms, and comparing those who wore the accelerometer (accelerometry participants) with those who did not (non‐participants). Descriptive characteristics were reported separately by sex, and Chi‐square and Wilcoxon signed‐rank tests were used to compare accelerometry participants and non‐participants. Post hoc analyses were done to explore non‐linearity: (1) stratifying by level of ADHD symptoms (below or above the clinical threshold) and (2) testing for an interaction between ADHD symptoms and ADHD tertiles. Since attenuation of associations may occur when a large proportion of the sample reports no symptoms, we conducted a post hoc analysis to explore this floor effect, excluding children with an ADHD symptom score of zero.

All analyses were done using the package “drgee” (Zetterqvist et al., 2016) in R, version 4.1.1 (R Core Team, 2025).

## RESULTS

A total of 1966 children (52.84% girls) were included in this study. The descriptive characteristics of the study population are summarized by sex in Table 1. Neuropsychiatric disorders were present in 1.37% of the population. On average, boys had a higher score of ADHD symptoms compared to girls. Boys and girls wore the accelerometer for around 800 min/day. Girls spent more time in LPA and less time in MVPA than boys (*P* < 0.001). SED was not significantly different between boys and girls. The associations between covariates and time spent in PA and SED are shown in Table S1.

**TABLE 1:** Descriptive characteristics of the study population by sex.

Characteristic	Girls (*n* = 1021)	Boys (*n* = 869)	*p*‐value
Neuropsychiatric disorders^a^, No. (%)	10 (0.9)	17 (1.8)	0.142
Score of ADHD symptoms, mean (SD)	7.8 (8.8)	11.2 (10.6)	<0.001
Accelerometry, average minutes/day, mean (SD)			
Wear time	803.1 (98.3)	805.5 (84.8)	0.018
Sedentary behavior (SED)	519.0 (103.1)	510.9 (91.3)	0.116
Light physical activity (LPA)	164.0 (26.4)	157.4 (27.6)	<0.001
Moderate‐to‐vigorous physical activity (MVPA)	120.1 (30.2)	137.3 (33.5)	<0.001

^a^ Neuropsychiatric disorders included neurological and psychiatric disorders other than ADHD.

### ADHD symptoms and time spent in PA and SED

Table 2 presents the associations between ADHD symptoms and PA and SED. In Model 1, adjusted for wear‐time, more ADHD symptoms were significantly associated with less time spent in SED (*β* = −0.32; 95% CI ‐0.59, −0.06) and more time spent in MVPA (*β* = 0.30; 95% CI 0.12, 0.47). The associations weakened and became non‐significant after adjusting for sex in Model 2. Significant interactions were found between ADHD symptoms and sex (all *p* < 0.05, see Table S2). Only hyperactivity/impulsivity symptoms, but not inattention, were significantly associated with PA and SED. In Model 1, hyperactivity/impulsivity was significantly associated with less time in SED (*β* = −0.93; 95% CI ‐1.60, −0.25), and more time in MVPA (*β* = 0.72, 95% CI 0.30, 1.15). The associations remained significant when adjusting for sex in Model 2. Significant interactions were found between hyperactivity/impulsivity and sex (all *p* < 0.05, see Table S3).

**TABLE 2:** Associations between ADHD symptoms and time spent in physical activity and sedentary behavior in the full cohort (*n* = 1936).

	Model 1		Model 2		Model 3	
	β (95% CI)	*p*‐value	β (95% CI)	*p*‐value	β (95% CI)	*p*‐value
Sedentary behavior (SED)						
ADHD symptoms	−0.32 (−0.59, −0.06)	0.015	−0.23 (−0.50, 0.03)	0.092	0.71 (−0.08, 1.50)	0.080
Inattention	0.23 (−0.38, 0.85)	0.454	0.36 (−0.26, 0.98)	0.255	0.06 (−1.81, 1.94)	0.946
Hyperactivity/impulsivity	−0.93 (−1.60, −0.25)	0.007	−0.87 (−1.54, −0.19)	0.011	1.41 (−0.65, 3.48)	0.180
Light physical activity (LPA)						
ADHD symptoms	0.02 (−0.11, 0.16)	0.692	0.08 (−0.05, 0.22)	0.250	−0.35 (−0.76, 0.06)	0.095
Inattention	−0.13 (−0.46, 0.19)	0.421	−0.06 (−0.39, 0.26)	0.660	0.16 (−0.83, 1.16)	0.746
Hyperactivity/impulsivity	0.20 (−0.16, 0.56)	0.271	0.24 (−0.11, 0.60)	0.189	−0.90 (−1.97, 0.17)	0.099
Moderate‐to‐vigorous physical activity (MVPA)						
ADHD symptoms	0.30 (0.12, 0.47)	<0.001	0.15 (−0.02, 0.32)	0.084	−0.36 (−0.86, 0.14)	0.162
Inattention	−0.10 (−0.49, 0.29)	0.617	−0.29 (−0.67, 0.08)	0.130	−0.23 (−1.45, 0.99)	0.721
Hyperactivity/impulsivity	0.72 (0.30, 1.15)	<0.001	0.62 (0.20, 1.04)	0.003	−0.51 (−1.85, 0.82)	0.468

*Note*: Model 1: adjusted for wear time. Model 2: adjusted for wear time and sex. Model 3: adjusted for wear time, sex, and interaction between sex × ADHD symptoms.

### Stratified analyses by sex

Since a significant interaction was observed between ADHD symptoms and sex, we performed a sex‐stratified analysis. As shown in Table 3, the associations between ADHD symptoms and time spent in PA and SED were only significant in girls. Among girls, more ADHD symptoms were significantly associated with less time spent in SED (*β* = −0.59; 95%CI ‐0.97, −0.20), and more time spent in LPA (*β* = 0.24; 95%CI 0.03, 0.45) and MVPA (*β* = 0.34; 95%CI 0.10, 0.58). Hyperactivity/impulsivity, but not inattention, was significantly associated with PA and SED in girls.

**TABLE 3:** Sex‐stratified associations between ADHD symptoms and time spent in physical activity and sedentary behavior.

Predictor	Girls (*n* = 1028)		Boys (*n* = 908)	
	β (95% CI)	*p*‐value	β (95% CI)	*p*‐value
Sedentary behavior (SED)				
ADHD symptoms	−0.59 (−0.97, −0.20)	0.002	0.04 (−0.31, 0.40)	0.818
Inattention	0.42 (−0.44, 1.29)	0.339	0.23 (−0.60, 1.08)	0.585
Hyperactivity/impulsivity	−1.68 (−2.66, −0.70)	<0.001	−0.16 (−1.07, 0.74)	0.727
Light physical activity (LPA)				
ADHD symptoms	0.24 (0.03, 0.45)	0.021	−0.04 (−0.22, 0.13)	0.644
Inattention	−0.12 (−0.58, 0.33)	0.592	0.02 (−0.42, 0.47)	0.914
Hyperactivity/impulsivity	0.64 (0.12, 1.16)	0.015	−0.11 (−0.58, 0.35)	0.633
Moderate‐to‐vigorous physical activity (MVPA)				
ADHD symptoms	0.34 (0.10, 0.58)	0.004	0.001 (−0.23, 0.23)	0.994
Inattention	−0.29 (−0.82, 0.22)	0.261	−0.25 (−0.81, 0.29)	0.357
Hyperactivity/impulsivity	1.03 (0.45, 1.61)	<0.001	0.27 (−0.32, 0.88)	0.370

*Note*: Model adjusted for wear time.

### Within‐twin pair analyses

The associations between ADHD symptoms and time spent in PA and SED stratified by zygosity and sex are presented in Table 4. Among DZ twin girls, only hyperactivity/impulsivity was significantly associated with both less time in SED and more time in LPA. The associations weakened and became non‐significant in MZ twin girls. Among boys, DZ twins showed a significant association between hyperactivity/impulsivity and both less time in SED and more time in MVPA. Inattention was significantly associated with more time in LPA. These associations became non‐significant in MZ twins.

**TABLE 4:** Within–twin pair associations between ADHD symptoms and time spent in physical activity and sedentary behavior stratified by sex and zygosity.

Predictor	Girls				Boys			
	Dizygotic twins (*n* = 288)		Monozygotic twins (*n* = 404)		Dizygotic twins (*n* = 230)		Monozygotic twins (*n* = 350)	
	β (95% CI)	*p*‐value	β (95% CI)	*p*‐value	β (95% CI)	*p*‐value	β (95% CI)	*p*‐value
Sedentary behavior (SED)								
ADHD symptoms	−0.43 (−0.92, 0.05)	0.084	−0.05 (−0.45, 0.31)	0.770	−0.89 (−1.43, −0.35)	0.001	0.29 (−0.19, 0.78)	0.238
Inattention	0.45 (−0.45, 1.37)	0.322	−0.17 (−1.10, 0.74)	0.706	−0.47 (−1.63, 0.69)	0.427	0.11 (−0.86, 1.09)	0.817
Hyperactivity/impulsivity	−1.50 (−2.56, −0.43)	0.005	0.10 (−1.21, 1.42)	0.880	−1.35 (−2.54, −0.16)	0.025	0.50 (−0.61, 1.63)	0.379
Light physical activity (LPA)								
ADHD symptoms	0.28 (−0.01, 0.57)	0.055	−0.01 (−0.23, 0.23)	0.990	0.51 (0.19, 0.83)	0.001	−0.12 (−0.44, 0.20)	0.471
Inattention	−0.35 (−0.85, 0.14)	0.168	0.30 (−0.18, 0.79)	0.218	0.82 (0.13, 1.51)	0.019	0.19 (−0.33, 0.72)	0.477
Hyperactivity/impulsivity	1.04 (0.38, 1.71)	0.002	−0.40 (−1.06, 0.24)	0.221	0.17 (−0.51, 0.86)	0.619	−0.49 (−1.30, 0.31)	0.232
Moderate‐to‐vigorous physical activity (MVPA)								
ADHD symptoms	0.14 (−0.18, 0.47)	0.379	0.05 (−0.18, 0.30)	0.639	0.37 (−0.01, 0.77)	0.058	−0.17 (−0.55, 0.21)	0.380
Inattention	−0.10 (−0.70, 0.48)	0.723	−0.12 (−0.69, 0.43)	0.653	−0.35 (−1.27, 0.56)	0.450	−0.30 (−1.10, 0.49)	0.451
Hyperactivity/impulsivity	0.45 (−0.20, 1.11)	0.179	0.30 (−0.48, 1.09)	0.447	1.17 (0.27, 2.08)	0.011	−0.01 (−0.89, 0.87)	0.979

*Note*: Model adjusted for wear time and conditional to the twin pair.

### Sensitivity and post hoc analyses

Compared to accelerometry participants (*n* = 2205), non‐participants (*n* = 3569) had a higher prevalence of neuropsychiatric disorders and lower maternal education level (Table S4). Non‐participant boys had a higher score in ADHD symptoms. According to the parental‐reported PA, accelerometry participants were more likely to participate in sports than non‐participants (*P* = 0.02 in boys, and *p* < 0.001 in girls). In the post hoc analyses, children with ADHD symptoms below the clinical threshold (*n* = 1784) showed associations that were in the same direction as when examining the full ADHD symptoms range. Children above the clinical threshold (*n* = 152) showed non‐significant associations (Tables S5 and S6). There were no significant interactions between ADHD symptoms and ADHD tertiles. When repeating the analyses excluding children with an ADHD score of zero (11.8%), the associations remained largely unchanged (Table S7).

## DISCUSSION

This study aimed to increase the understanding of the associations between ADHD symptoms and time spent in PA and SED during childhood. In the full cohort, we observed that ADHD symptoms were significantly associated with less time spent in SED and more time spent in MVPA. The associations appear to be mainly driven by hyperactivity/impulsivity symptoms and were significant only among girls. In the within‐twin pair analyses, the associations were significant in DZ twins but became non‐significant in MZ twins, suggesting that shared familial factors, including genetic factors, explain these associations.

Our results provide new insights into the relationship between ADHD symptoms and time spent in PA and SED. Previous studies in children with ADHD diagnosis have reported an association between ADHD diagnosis and more time spent in MVPA (Lin et al., 2013; Sempere‐Tortosa et al., 2021; Villalba‐Heredia et al., 2022). Besides finding a similar association, with higher ADHD symptoms associated with more time in MVPA, we found that ADHD symptoms were associated with less time in SED. Furthermore, our findings suggests that these associations extend to subthreshold presentations of ADHD symptoms, since, in a post hoc analysis, the associations were not only driven by children above the clinical threshold. Additionally, we found no evidence of non‐linearity. Interaction analyses based on ADHD symptom tertiles did not indicate significant differences in the linear slopes across symptom levels, suggesting that the association was largely consistent across the full ADHD score distribution.

This study further extends existing knowledge by showing that the associations depend on symptom type, with the hyperactivity/impulsivity dimension mainly driving them. A similar trend has been observed in a population‐based study using self‐reported PA; hyperactivity symptoms in childhood predicted more PA in adolescence, whereas inattention symptoms predicted less PA (Selinus et al., 2021). Accelerometry‐based studies have rarely explored these associations, with only one study reporting no differences in PA behavior between ADHD subtypes (Villalba‐Heredia et al., 2022). Although our findings might differ from Villalba‐Heredia et al. due to differences in study populations, as they only include children with an ADHD diagnosis, this might also reflect differences in data processing. The accelerometry cut‐off points used influence results and hinder comparisons of findings across studies (Machado‐Rodrigues et al., 2025). Additionally, shorter epochs capture more detailed PA data. Since Villalba‐Heredia et al. used longer epochs (10‐s vs. 3‐s in our study), their results may have a stronger averaging effect, obscuring relevant details (Sievänen & Kujala, 2017). Although our results align with other accelerometry studies, self‐reported PA studies have repeatedly found that ADHD is associated with lower PA engagement (Holton & Nigg, 2020; Khalife et al., 2014; Kim et al., 2011). These findings are not necessarily contradictory, as questionnaires and accelerometers capture different dimensions of PA. Questionnaires tend to assess structured activities (e.g., sports), whereas accelerometers also capture unstructured activities (e.g., play and active transport), which may be more difficult to recall and report. This is particularly relevant in children with ADHD, as research suggests that they tend to engage more in PA in unstructured settings (Lin et al., 2013). In addition, it could be that questionnaire‐based studies are affected by recall bias, as individuals with psychopathology tend to under‐ or overestimate their PA (Collins et al., 2025). Considering this, accelerometry might be a more suitable alternative to measure PA in association with ADHD.

Sex and genetic factors may have an important role in the association between ADHD symptoms and time spent in PA and SED. An interaction between ADHD symptoms and sex was found, with the observed associations being significant only among girls. This could mean that hyperactivity/impulsivity symptoms have a greater role in girls' PA than in boys. However, this association can also be what could be called a social artifact. If parents expect boys to have higher MVPA than girls, they may overreport hyperactivity in girls with high MVPA. Furthermore, our findings suggest that the associations observed are partially due to shared genetic factors. It seems plausible that the genes involved in hyperactivity symptoms, such as restlessness, may also influence the time spent in PA. Future studies are needed to examine and quantify the potential genetic overlap between ADHD symptoms and time in PA and SED.

PA is an important determinant of health. Higher PA has been associated with a lower risk of cardiovascular diseases and better brain health (Ashdown‐Franks et al., 2020; Pozuelo‐Carrascosa et al., 2018; Wiklund et al., 2024). Monitoring PA levels in populations at increased risk of adverse health outcomes, such as individuals with ADHD symptoms, is therefore important (Du Rietz et al., 2024; Stott et al., 2026). We found that at age nine, ADHD symptoms are not associated with less time in PA, but rather the opposite is true. These findings challenge the assumption that ADHD symptoms are universally associated with poor lifestyle factors. Additionally, our findings highlight the importance of designing targeted public health interventions that take factors like sex and the type of ADHD symptoms into consideration. Longitudinal studies are needed to determine whether these associations persist into adolescence and adulthood and to examine whether early movement behaviors influence later cardiovascular and mental health outcomes among individuals with ADHD symptoms.

The magnitude of associations was modest. In girls, a one‐point increase in hyperactivity/impulsivity symptoms corresponded to approximately one additional minute of MVPA and 1.7 fewer minutes of SED per day. In the post hoc analysis excluding children with an ADHD symptoms score of zero, the estimates remained largely unchanged, suggesting that the findings were not attenuated by a floor effect. However, small effects are expected since this is a population‐based study and ADHD symptoms represent only one of many determinants of PA and SED. The consistent direction of the associations suggests that children with hyperactivity/impulsivity symptoms may be predisposed to spend more time engaging in PA. If this is the case, it underscores the importance of early interventions to reinforce this behavior, as even small shifts in MVPA at the population level may translate into meaningful health benefits over time.

The findings should be interpreted considering the study's strengths and limitations. One strength of this study is the use of a large, population‐based twin sample, which allowed subgroup analyses and adjustment for familial confounding. Furthermore, using triaxial accelerometry enabled us to capture estimates that are not subject to recall bias and are less susceptible to social desirability bias.

This study had a few limitations. First, the cross‐sectional design limits conclusions around directionality. Second, 30% of children agreed to wear the accelerometer. Accelerometry participants had lower ADHD symptoms and reported higher PA than non‐participants, which may have reduced our power and sample heterogeneity. Third, although based on the items included in the DSM‐5 to assess ADHD, the tool used to measure ADHD symptoms had unknown psychometric properties. This may have introduced measurement error and increased the risk of type II error, attenuating the observed associations through dilution and reducing statistical power. Consequently, our estimates may underestimate the true magnitude of the associations. Fourth, the young age of the sample limits the number of children who have developed ADHD symptoms and the number of those diagnosed with neuropsychiatric disorders. Residual confounding is possible since we could not adjust for the use of ADHD medication. In addition, accelerometers cannot capture water‐based activities and underestimate activities with little hip movement (e.g., bicycling), leading to an underestimation of the associations with LPA and MVPA. Furthermore, we could not account for the interrelated nature of the PA and SED variables. Our sample included only twins, since having siblings may increase time spent in MVPA (Kracht & Sisson, 2018); effect sizes might differ among those without siblings. Finally, the study population was born in Sweden and had high maternal education, potentially limiting generalizability to groups with different social and cultural contexts.

## CONCLUSION

Our findings suggest that children with ADHD symptoms might have different PA and SED depending on sex and the type of symptoms. Among girls, hyperactivity/impulsivity symptoms were continuously associated with less time spent in SED and more time spent in MVPA. These associations seemed to be explained by shared familial factors, including genetics. Further longitudinal research is needed to corroborate these results.

## AUTHOR CONTRIBUTIONS

**Narda Ontiveros**: Conceptualization; methodology; investigation; formal analysis; writing—original draft. **Björg Helgadottir**: Data Curation; formal analysis; methodology; writing—review and editing. **Örjan Ekblom**: Conceptualization; supervision; funding acquisition; methodology; writing—review and editing. **Anna Ohlis**: Supervision; writing—review and editing. **Aylin Mehren**: Supervision; writing—review and editing. **Camilla A. Wiklund**: Conceptualization; methodology; supervision; writing—review and editing.

## CONFLICT OF INTEREST STATEMENT

The authors declare no conflicts of interest.

## ETHICAL CONSIDERATIONS

This study was approved by the Swedish Ethical Review Authority (approval date: 2016‐12‐31, reference numbers 2016/2135‐31 and 2020‐00594). This study used data from the Child and Adolescent Twin Study in Sweden, parents provided written informed consent for participation in the study. Data have been pseudonymized and stored in a secure environment with restricted access. Individuals did not receive any direct benefit, nor were they exposed to a particular risk by being included in this study.

## Supporting information

Tables S1–S7

## Data Availability

The data used in this study were extracted from the Swedish Twin Registry. Researchers may apply to the Swedish Ethical Review Authority and the Swedish Twin Registry for access to the data.
