# Supplementary material for: Association between ADHD symptoms and device‐measured physical activity and sedentary behavior in childhood: A population‐based twin study
Source: JCPP Adv. 2026 Jun 9:e70141. Online ahead of print. doi: 10.1002/jcv2.70141 (PMC13339111; doi:10.1002/jcv2.70141)
Supplement: Supplementary file 1 — Tables S1–S7 [file JCV2-9999-e70141-s001.docx]

**Association between ADHD symptoms and device-measured physical activity and sedentary behavior in childhood: a population-based twin study**

**Supporting information**

|  | SED | | LPA | | MVPA | |
| --- | --- | --- | --- | --- | --- | --- |
| Covariates | $\boldsymbol{\beta}$ **(95% CI)** | **p-value** | $\boldsymbol{\beta}$ **(95% CI)** | **p-value** | $\boldsymbol{\beta}$ **(95% CI)** | **p-value** |
| Girls (vs boys) | 8.41 (-1.23, 17.51) | 0.088 | 6.66 (3.85, 9.38) | <0.001 | -17.21 (-20.57, -13.84) | <0.001 |
| Neuropsychiatric disorders^a^ (vs no) | 17.63 (-17.47, 52.73) | 0.325 | -19.62 (-38.20, -1.05) | 0.038 | -19.11 (-35.87, -2.35) | 0.025 |
| ^a^Neuropsychiatric disorders included neurological and psychiatric disorders other than ADHD. | | | | | | |

**Table S1.** Associations between covariates and time spent in physical activity and sedentary behavior (n=1,966).

**Table S2.** Associations between ADHD symptoms and time spent in physical activity and sedentary behavior in the full cohort (n=1,936).

|  | Model 1 | | Model 2 | | Model 3 | |
| --- | --- | --- | --- | --- | --- | --- |
|  | $\boldsymbol{\beta}$ **(95% CI)** | **p-value** | $\boldsymbol{\beta}$ **(95% CI)** | **p-value** | $\boldsymbol{\beta}$ **(95% CI)** | **p-value** |
|  | **Sedentary behavior (SED)** | | | | | |
| Intercept | -212.41 (-243.93, -180.89) | <0.001 | -231.19 (-264.14, -198.24) | <0.001 | -240.12 (-273.80, -206.44) | <0.001 |
| ADHD symptoms | -0.32 (-0.59, -0.06) | 0.015 | -0.23 (-0.50, 0.03) | 0.092 | 0.71 (-0.08, 1.50) | 0.080 |
| Wear time | 0.90 (0.86, 0.94) | <0.001 | 0.90 (0.87, 0.94) | <0.001 | 0.90 (0.87, 0.94) | <0.001 |
| Sex (boys=0, girls=1) |  |  | 10.83 (5.41, 16.25) | <0.001 | 16.83 (9.90, 23.76) | <0.001 |
| Sex*ADHD symptoms |  |  |  |  | -0.65 (-1.17, -0.14) | 0.012 |
|  | **Light physical activity (LPA)** | | | | | |
| Intercept | 117.79 (100.89, 134.68) | <0.001 | 106.78 (88.50, 125.07) | <0.001 | 110.87 (92.19, 129.56) | <0.001 |
| ADHD symptoms | 0.02 (-0.11, 0.16) | 0.692 | 0.08 (-0.05, 0.22) | 0.250 | -0.35 (-0.76, 0.06) | 0.095 |
| Wear time | 0.05 (0.03, 0.07) | <0.001 | 0.05 (0.03, 0.07) | <0.001 | 0.05 (0.03, 0.07) | <0.001 |
| Sex (boys=0, girls=1) |  |  | 6.34 (3.57, 9.12) | <0.001 | 3.60 (0.04, 7.15) | 0.046 |
| Sex*ADHD symptoms |  |  |  |  | 0.30 (0.03, 0.57) | 0.029 |
|  | **Moderate-to-vigorous physical activity (MVPA)** | | | | | |
| Intercept | 94.62 (77.09, 112.15) | <0.001 | 124.40 (106.78, 142.03) | <0.001 | 129.24 (111.20, 147.29) | <0.001 |
| ADHD symptoms | 0.30 (0.12, 0.47) | <0.001 | 0.15 (-0.02, 0.32) | 0.084 | -0.36 (-0.86, 0.14) | 0.162 |
| Wear time | 0.03 (0.01, 0.06) | <0.001 | 0.03 (0.01, 0.05) | <0.001 | 0.03 (0.01, 0.05) | <0.001 |
| Sex (boys=0, girls=1) |  |  | -17.18 (-20.59, -13.77) | <0.001 | -20.43 (-24.92, -15.94) | <0.001 |
| Sex*ADHD symptoms |  |  |  |  | 0.35 (0.03, 0.67) | 0.029 |

**Table S3.** Association between inattention, hyperactivity/impulsivity, and time spent in physical activity and sedentary behavior in the full cohort (n=1,936).

|  | Model 1 | | Model 2 | | Model 3 | |
| --- | --- | --- | --- | --- | --- | --- |
|  | $\boldsymbol{\beta}$ **(95% CI)** | **p-value** | $\boldsymbol{\beta}$ **(95% CI)** | **p-value** | $\boldsymbol{\beta}$ **(95% CI)** | **p-value** |
|  | **Sedentary behavior (SED)** | | | | | |
| Intercept | -212.25 (-243.89, -180.62) | <0.001 | -231.20 (-264.24, -198.16) | <0.001 | -240.32 (-274.03, -206.61) | <0.001 |
| Inattention | 0.23 (-0.38, 0.85) | 0.454 | 0.36 (-0.26, 0.98) | 0.255 | 0.06 (-1.81, 1.94) | 0.946 |
| Hyperactivity/  impulsivity | -0.93 (-1.60, -0.25) | 0.007 | -0.87 (-1.54, -0.19) | 0.011 | 1.41 (-0.65, 3.48) | 0.180 |
| Wear time | 0.90 (0.86, 0.94) | <0.001 | 0.90 (0.87, 0.94) | <0.001 | 0.90 (0.87, 0.94) | <0.001 |
| Sex (boys=0, girls=1) |  |  | 10.94 (5.52, 16.35) | <0.001 | 16.97 (10.03, 23.90) | <0.001 |
| Sex*inattention |  |  |  |  | 0.17 (-1.02, 1.37) | 0.776 |
| Sex*hyperactivity/ impulsivity |  |  |  |  | -1.55 (-2.89, -0.21) | 0.023 |
|  | **Light physical activity (LPA)** | | | | | |
| Intercept | 117.74 (100.81, 134.67) | <0.001 | 106.79 (88.49, 125.09) | <0.001 | 110.96 (92.28, 129.63) | <0.001 |
| Inattention | -0.13 (-0.46, 0.19) | 0.421 | -0.06 (-0.39, 0.26) | 0.660 | 0.16 (-0.83, 1.16) | 0.746 |
| Hyperactivity/  impulsivity | 0.20 (-0.16, 0.56) | 0.271 | 0.24 (-0.11, 0.60) | 0.189 | -0.90 (-1.97, 0.17) | 0.099 |
| Wear time | 0.05 (0.03, 0.07) | <0.001 | 0.05 (0.03, 0.07) | <0.001 | 0.05 (0.03, 0.07) | <0.001 |
| Sex (boys=0, girls=1) |  |  | 6.32 (3.54, 9.09) | <0.001 | 3.57 (0.01, 7.12) | 0.044 |
| Sex*inattention |  |  |  |  | -0.14 (-0.77, 0.49) | 0.660 |
| Sex*hyperactivity/ impulsivity |  |  |  |  | 0.77 (0.07, 1.47) | 0.030 |
|  | **Moderate-to-vigorous physical activity (MVPA)** | | | | | |
| Intercept | 94.51 (76.92, 112.10) | <0.001 | 124.41 (106.72, 142.10) | <0.001 | 129.36 (111.29, 147.44) | <0.001 |
| Inattention | -0.10 (-0.49, 0.29) | 0.617 | -0.29 (-0.67, 0.08) | 0.130 | -0.23 (-1.45, 0.99) | 0.721 |
| Hyperactivity/  impulsivity | 0.72 (0.30, 1.15) | <0.001 | 0.62 (0.20, 1.04) | 0.003 | -0.51 (-1.85, 0.82) | 0.468 |
| Wear time | 0.03 (0.01, 0.06) | <0.001 | 0.03 (0.01, 0.05) | <0.001 | 0.03 (0.01, 0.05) | <0.001 |
| Sex (boys=0, girls=1) |  |  | -17.26 (-20.66, -13.85) | <0.001 | -20.54 (-25.03, -16.05) | <0.001 |
| Sex*inattention |  |  |  |  | -0.03 (-0.78, 0.72) | 0.935 |
| Sex*hyperactivity/ impulsivity |  |  |  |  | 0.77 (-0.05, 1.61) | 0.068 |

**Table S4.** Descriptive characteristics of accelerometry participants and non-participants, stratified by sex.

|  | **Girls** | | | | **Boys** | | | |
| --- | --- | --- | --- | --- | --- | --- | --- | --- |
| **Characteristic** | **All (n=2,957)** | **Participants (n=1,164)** | **Non-participants (n=1,793)** | **P-value** | **All (n=2,817)** | **Participants (n=1,041)** | **Non-participants (n=1,776)** | **P-value** |
| Neuropsychiatric disorders^a^, No. (%) | 59(2.0) | 11(0.9) | 48(2.7) | 0.002 | 76(2.71) | 20(1.9) | 56(3.1) | 0.064 |
| ADHD symptoms, mean (SD) | 8.3(9.5) | 7.8(8.9) | 8.6(9.8) | 0.097 | 11.7(11.2) | 11.06(10.5) | 12.1(11.5) | 0.053 |
| Maternal education level | | | | | | | | |
| Elementary school (<10 years) | 52(1.8) | 16(1.4) | 36(2.1) | 0.018 | 38(1.4) | 14(1.4) | 24(1.4) | <0.001 |
| Upper secondary school (10-12 years) | 783(27.6) | 282(25.1) | 501(29.2) |  | 829(30.9) | 263(26.3) | 566(33.6) |  |
| University (>12 years) | 2002(70.6) | 824(73.5) | 1178(68.7) |  | 1820(67.7) | 723 (72.3) | 1097(65.0) |  |
| Parental-report physical activity, No. (%) | | | | | | | | |
| *“How much does your child move?”* | | | | | | | | |
| Never so much that they get out of breath | 143(4.8) | 34(2.9) | 109(6.1) | <0.001 | 112(4.0) | 20(1.9) | 92(5.2) | <0.001 |
| S/he gets out of breath once a week | 639(21.7) | 215(18.5) | 424(23.8) |  | 439(15.6) | 142(13.7) | 297(16.8) |  |
| S/he gets out of breath several times a week | 1536(52.1) | 623(53.6) | 913(51.1) |  | 1177(41.8) | 428(41.1) | 749(42.2) |  |
| S/he gets out of breath almost every day | 631(21.4) | 291(25.0) | 340(19.0) |  | 1085(38.6) | 451(43.3) | 634(35.8) |  |
| *“Does your child exercise or play sports in his/her free time?”* | | | | | | | | |
| Never/rarely | 547(18.5) | 173(14.9) | 374(20.9) | <0.001 | 520(18.5) | 164(15.8) | 356(20.1) | 0.022 |
| Once a week | 733(24.8) | 251(21.6) | 482(26.9) |  | 493(17.5) | 177(17.0) | 316(17.8) |  |
| Several times a week | 1607(54.4) | 708(60.8) | 899(50.1) |  | 1678(59.6) | 647(62.3) | 1031(58.0) |  |
| Almost daily | 69(2.3) | 32(2.7) | 37(2.1) |  | 124(4.4) | 51(4.9) | 73(4.1) |  |
| ^a^Neuropsychiatric disorders included neurological and psychiatric disorders other than ADHD | | | | | | | | |

**Table S5.** Associations between ADHD symptoms and time spent in physical activity and sedentary behavior by level of symptoms using clinical thresholds in girls.

| Predictor | ADHD symptoms below clinical threshold (n=969) | | ADHD symptoms above clinical threshold (n=59) | |
| --- | --- | --- | --- | --- |
|  | $\boldsymbol{\beta}$ **(95% CI)** | **p-value** | $\boldsymbol{\beta}$ **(95% CI)** | **p-value** |
|  | **Sedentary behavior (SED)** | | | |
| ADHD symptoms | -0.79 (-138, -0.21) | 0.007 | -0.27 (-1.81, 1.25) | 0.723 |
| Inattention | 0.47 (-0.64, 1.58) | 0.405 | 0.21 (-2.64, 3.07) | 0.882 |
| Hyperactivity/ impulsivity | -2.04 (-3.21, -0.87) | <0.001 | -0.74 (-2.71, 1.21) | 0.455 |
|  | **Light physical activity (LPA)** | | | |
| ADHD symptoms | 0.43 (0.13, 0.73) | 0.004 | 0.09 (-0.84, 1.02) | 0.848 |
| Inattention | 0.18 (-0.41, 0.78) | 0.544 | -0.62 (-2.22, 0.96) | 0.440 |
| Hyperactivity/ impulsivity | 0.68 (0.10, 1.25) | 0.019 | 0.77 (-0.40, 1.96) | 0.197 |
|  | **Moderate-to-vigorous physical activity (MVPA)** | | | |
| ADHD symptoms | 0.36 (-0.01, 0.74) | 0.060 | 0.18 (-0.58, 0.95) | 0.634 |
| Inattention | -0.65 (-1.31, -0.01) | 0.047 | 0.41 (-1.04, 1.86) | 0.578 |
| Hyperactivity/ impulsivity | 1.36 (0.61, 2.10) | <0.001 | -0.02 (-1.02, 0.96) | 0.955 |
| Models were adjusted for wear time.  Clinical threshold for ADHD symptoms= at least 6 symptoms scored as "often" or “very often” (scored 2 or 3) to either the inattention or hyperactivity/impulsivity items or both. | | | | |

**Table S6.** Associations between ADHD symptoms and time spent in physical activity and sedentary behavior by level of symptoms using clinical thresholds in boys.

| Predictor | ADHD symptoms below clinical threshold (n=815) | | ADHD symptoms above clinical threshold (n=93) | |
| --- | --- | --- | --- | --- |
|  | $\boldsymbol{\beta}$ **(95% CI)** | **p-value** | $\boldsymbol{\beta}$ **(95% CI)** | **p-value** |
|  | **Sedentary behavior (SED)** | | | |
| ADHD symptoms | -0.48 (-0.99, 0.01) | 0.058 | 0.51 (-0.94, 1.98) | 0.488 |
| Inattention | -0.18 (-1-27, 0.90) | 0.740 | 0.76 (-0.69, 2.22) | 0.302 |
| Hyperactivity/ impulsivity | -0.81 (-2.00, 0.37) | 0.180 | 0.32 (-1.88, 2.54) | 0.771 |
|  | **Light physical activity (LPA)** | | | |
| ADHD symptoms | 0.20 (-0.06, 0.47) | 0.131 | -0.07 (-0.81, 0.66) | 0.845 |
| Inattention | 0.07 (-0.47, 0.61) | 0.790 | 0.27 (-0.84, 1.39) | 0.631 |
| Hyperactivity/ impulsivity | 0.35 (-0.23, 0.93) | 0.237 | -0.33 (-1.36, 0.69) | 0.519 |
|  | **Moderate-to-vigorous physical activity (MVPA)** | | | |
| ADHD symptoms | 0.28 (-0.03, 0.60) | 0.086 | -0.44 (-1.49, 0.60) | 0.405 |
| Inattention | 0.11 (-0.59, 0.81) | 0.756 | -1.04 (-2.35, 0.26) | 0.118 |
| Hyperactivity/ impulsivity | 0.46 (-0.29, 1.22) | 0.232 | 0.01 (-1.44, 1.45) | 0.990 |
| Models were adjusted for wear time.  Clinical threshold for ADHD symptoms= at least 6 symptoms scored as "often" or “very often” (scored 2 or 3) to either the inattention or hyperactivity/impulsivity items or both. | | | | |

| Predictor | Girls (n=873) | | Boys (n=834) | |
| --- | --- | --- | --- | --- |
|  | $\boldsymbol{\beta}$ **(95% CI)** | **p-value** | $\boldsymbol{\beta}$ **(95% CI)** | **p-value** |
|  | **Sedentary behavior (SED)** | | | |
| ADHD symptoms | -0.57 (-0.97, -0.17) | 0.005 | 0.01 (-0.36, 0.38) | 0.966 |
| Inattention | 0.43 (-0.44, 1.30) | 0.330 | 0.20 (-0.64, 1.06) | 0.634 |
| Hyperactivity/ impulsivity | -1.66 (-2.65, -0.67) | <0.001 | -0.20 (-1.12, 0.71) | 0.667 |
|  | **Light physical activity (LPA)** | | | |
| ADHD symptoms | 0.22 (0.01, 0.44) | 0.047 | -0.05 (-0.24, 0.13) | 0.580 |
| Inattention | -0.14 (-0.60, 0.31) | 0.543 | 0.01 (-0.44, 0.46) | 0.971 |
| Hyperactivity/ impulsivity | 0.62 (0.09, 1.15) | 0.021 | -0.11 (-0.58, 0.35) | 0.624 |
|  | **Moderate-to-vigorous physical activity (MVPA)** | | | |
| ADHD symptoms | 0.35 (0.11, 0.58) | 0.003 | 0.04 (-0.19, 0.28) | 0.718 |
| Inattention | -0.29 (-0.81, 0.23) | 0.275 | -0.21 (-0.77, 0.34) | 0.451 |
| Hyperactivity/ impulsivity | 1.04 (0.46, 1.61) | <0.001 | 0.31 (-0.29, 0.93) | 0.305 |
| Models were adjusted for wear time. | | | | |

**Table S7.** Sex-stratified associations between ADHD symptoms and time spent in physical activity and sedentary behavior, excluding children without ADHD symptoms (ADHD symptoms score = 0).
